# The role of surgery type in postoperative atrial fibrillation and in-hospital mortality in esophageal cancer patients with preserved left ventricular ejection fraction

**DOI:** 10.1186/s12957-020-02011-6

**Published:** 2020-09-11

**Authors:** Laite Chen, Lu Zhang, Lu Shi, Guosheng Fu, Chenyang Jiang

**Affiliations:** 1 Department of Cardiology of Sir Run Run Shaw Hospital, Zhejiang University School of Medicine, No. 3 Qingchun East Road, Zhejiang, 310000 Hangzhou China; 2 Department of Public Health Sciences, Clemson University, Clemson, SC USA

**Keywords:** Esophagectomy, Postoperative atrial fibrillation, Minimally invasive surgery, Esophageal cancer, Mortality

## Abstract

**Background:**

Postoperative atrial fibrillation (POAF) is one of the most common complications of esophagectomy, which may extend the inpatient hospital stay. Minimally invasive esophagectomy (MIE) has been increasingly used in clinical practice; however, its POAF risk and short-term mortality remain unclear. This study aimed to examine the POAF risk and in-hospital mortality rate between patients receiving MIE and open esophagectomy (OE).

**Methods:**

Esophageal cancer patients who underwent MIE or OE from a retrospective cohort study were evaluated. A multivariate logistic regression model was built to assess the associations between esophagectomy (MIE vs. OE) and various outcomes (POAF, in-hospital mortality). Covariates included age, sex, body mass index, neoadjuvant therapy, tumor stage, surgery incision type, comorbidities, cardia conditions, peri-operative medication, and complications.

**Results:**

Of the 484 patients with esophageal cancer, 63 received MIE. A total of 53 patients developed POAF. Compared to patients receiving OE, MIE patients had 81% reduced odds of POAF (adjusted odds ratio [aOR] 0.185, 95% CI 0.039–0.887, *P* = 0.035). No statistically significant association was found for in-hospital mortality (aOR 0.709, 95% CI 0.114–4.409, *P* = 0.712).

**Conclusions:**

MIE is associated with a lower risk of POAF, compared to traditional surgery. No significant short-term survival benefit was found for MIE.

## Introduction

Postoperative atrial fibrillation (POAF) is one of the most common complications of esophagectomy that often occur within 3 days after esophagectomy [1]. Studies showed that POAF could lead to a longer hospital stay and a greater incidence of other complications [2]. POAF risk is higher among patients who are older [3], racial/ethnic minorities [4], taking perioperative medications such as β-blocker [5], and having hypertension [6] or operative trauma [7].

In recent decades, minimally invasive esophagectomy (MIE) has become the preferred procedure for esophagectomy due to its faster recovery and improved quality of life [8–10]. However, the association between MIE and the risk of POAF has not been well documented. For example, several studies investigating postoperative complications of MIE grouped POAF with other cardiovascular complications together [11–13]. Other studies evaluating POAF risk as a single outcome yielded inconsistent results [14–17]: while one study reported decreased POAF risk after MIE [18], other studies did not find any association between MIE and POAF [16, 19]. Another gap in the literature is the lack of consistent short-term mortality rate data after MIE. Several studies concluded that MIE had favorable outcomes of in-hospital mortality [11, 20] while other studies suggested a non-significant result [21, 22]. It is important to investigate the POAF risk and short-term mortality after MIE.

In the present study, we aimed to examine the association of esophagectomy surgery type (MIE vs. open esophagectomy [OE]) with POAF risk, and in-hospital mortality.

## Materials and methods

### Study population

Stage 0, I, or II esophageal cancer patients who underwent MIE or OE between January 2005 and April 2012 in Asan Medical Center, University of Ulsan College of Medicine, Seoul, South Korea, were identified, among which, patients with preoperative records or history of atrial fibrillation, atrial flutter, paced rhythm, and mid-range (40–49%) or reduced (< 40%) left ventricular ejection fraction (LVEF) [23] were excluded from this study (*n* = 8). The dataset was obtained from an online platform “FigShare” which provides a public accessible de-identified dataset (https://figshare.com/articles/Association_between_Postoperatively_Developed_Atrial_Fibrillation_and_Long-Term_Mortality_after_Esophagectomy_in_Esophageal_Cancer_Patients_An_Observational_Study/3306883) [24]. FigShare is a repository where users can make all of their research outputs available in a citable, shareable, and discoverable manner. Data from FigShare is available under the Creative Commons Attribution License (CCAL) which allows anyone to download, reuse, reprint, modify, distribute, and/or copy data from FigShare [25].

### Variables

#### Exposure and outcome

Exposure variable is surgery type: MIE vs. OE. MIE included thoracoscopy, thoracoscopy combined with laparoscopy, or robot-assisted thoracoscopy. The primary outcome was POAF, defined as the newly developed AF after esophagectomy prior to discharge that required therapy irrespective of the AF duration. Treatments for POAF included electrical and medication cardioversion (300 mg amiodarone of intravenous bolus followed by 1500 mg/day for 24 h) [24]. The secondary outcome was in-hospital mortality (mortality during hospital stay or within 30 days after surgery).

### Covariates

Previous studies have shown that demographic factors [3], comorbidity and perioperative medication [26–28], cardiac assessment [29], and oncological characteristics [17] were associated with the incidence of POAF. Therefore, in the multivariable analysis of POAF, covariates included age (< 60 and ≥ 60 years), sex, body mass index (BMI < 25 kg/m^2^ and ≥ 25 kg/m^2^), cancer stage (0, I, and II), diabetes mellitus (DM), hypertension (HTN), type of incision (Ivor Lewis, McKeown, and transhiatal), preoperative heart rate (HR) (by quartiles), preoperative left ventricular ejection fraction (LVEF) (by quartiles), β-blocker use, diuretics use, and neoadjuvant therapy. In the Ivor Lewis esophagectomy, a laparotomy was performed with a gastroepiploic artery reserved, followed by a right thoracotomy, resection of the esophagus. In the transhiatal esophagectomy, the esophageal tumor was removed through an abdominal incision, without thoracotomy, and a left neck incision. McKeown esophagectomy included thoracic esophageal mobilization, lymph node dissection, ligate thoracic duct (thoracoscopic or open), abdominal exploration (laparoscopic or open), stomach mobilization, lymph node dissection, feeding jejunostomy, and left cervical incision for anastomosis.

In addition, previous research reported that perioperative complications (anastomotic leak, pneumonia, sepsis) were associated with POAF [19, 30, 31]. However, because the timing of the occurrences of POAF and the complications were not well documented in this dataset, we did not control for these complications as confounders in the main analysis of POAF but conducted a sensitivity analysis with adjustment of the three complications.

For short-term survival (in-hospital mortality), previous studies suggested that demographic factors [32], medical history [33], complications [34], and oncological characteristics [35] are associated with mortality rate of cancer patients. Thus, multivariable analysis of in-hospital mortality adjusted for all the covariates in POAF analysis except preoperative HR and LVEF, and additionally adjusted for postoperative complications including pneumonia, anastomotic leak, sepsis, and acute kidney injury.

### Statistical analysis

Baseline characteristics were compared between MIE and OE, using descriptive statistics. Categorical variables were demonstrated in frequency and compared by the chi-square test. Multivariable logistic regression was used to examine the association of surgery type with POAF, in-hospital mortality, adjusting for potential confounders. All analyses were processed using Stata (Stata Statistical Software, version 16; StataCorp LP). Significance was set at *P* < 0.05.

## Results

Of 482 patients, 419 patients underwent OE and 63 received MIE. The majority of the patients were male, over 60 years old, with BMI < 25 kg/m^2^, without DM or HTN comorbidities, without β-blocker or diuretics use, and not having perioperative complications of pneumonia or anastomotic leak. Compared to OE patients, MIE patients were more likely to have DM, anastomotic leak from surgery, and McKeown incision type. In total, 53 (11.0%) patients developed POAF, including 48 (11.5%) among OE patients and 5 (7.9%) among MIE patients (*P* = 0.519). There were five cases of POAF associated with anastomotic leak. Large amounts of patients (*n* = 344) in this dataset underwent intrathoracic anastomosis, 21 of these had anastomotic leak. Among patients who underwent intrathoracic anastomosis and subsequently complicated with leak, four of them developed POAF. Among 17 (3.5%) patients dying in the hospital or within 30 days after surgery, 14 (3.3%) were OE patients and 3 (4.8%) were MIE patients (*P* = 0.476) (Table 1).

**Table 1:** Characteristics of patients undergoing open esophagectomy and minimally invasive esophagectomy (data are presented as *n* (%))

Variables	Total (*N* = 482)	OE (*N* = 419)	MIE (*N* = 63)	*P* value
Demographics				
Age ≥ 60 years	318 (66.0)	271 (64.7)	47 (74.6)	0.121
Sex (male)	451 (93.6)	391 (93.3)	60 (95.2)	0.784
BMI ≥ 25 kg/m^2^	125 (25.9)	106 (25.3)	19 (30.2)	0.412
Medical history				
DM	81 (16.8)	63 (15.0)	18 (28.6)	0.007
HTN	160 (33.2)	139 (33.2)	21 (33.3)	0.980
Cardiac condition				
HR				0.256
≤ 51	138 (28.6)	115 (27.4)	23 (36.5)	
< 51–74	119 (24.7)	102 (24.3)	17 (27.0)	
< 74–99	116 (24.1)	102 (24.3)	14 (22.2)	
> 99	109 (22.6)	100 (23.9)	9 (14.3)	
LVEF				0.333
≤ 59	97 (26.4)	86 (27.0)	11 (22.0)	
< 59–62	107 (29.1)	91 (28.6)	16 (32.0)	
< 62–65	88 (23.9)	72 (22.6)	16 (32.0)	
> 65	76 (20.7)	69 (21.7)	7 (14.0)	
Medications				
β-Blocker use	30 (6.2)	24 (5.7)	6 (9.5)	0.245
Diuretics use	39 (8.1)	33 (7.9)	6 (9.5)	0.655
Cancer related				
Neoadjuvant therapy	189 (39.2)	183 (43.7)	6 (9.5)	< 0.001
Pathologic stage of cancer				0.006
Stage 0	111 (23.0)	105 (25.1)	6 (9.5)	
Stages I and II	371 (77.0)	314 (74.9)	57 (90.5)	
Incision				< 0.001
Ivor Lewis	344 (71.4)	339 (80.9)	5 (7.9)	
McKeown	134 (27.8)	76 (18.1)	58 (92.1)	
Transhiatal	4 (0.8)	4 (1.0)	0 (0.0)	
Complications				
Pneumonia	75 (15.6)	64 (15.3)	11 (17.5)	0.655
Anastomotic leak	30 (6.2)	22 (5.3)	8 (12.7)	0.023
Sepsis	78 (16.2)	66 (15.8)	12 (19.0)	0.508
Acute kidney injury	169 (35.1)	147 (35.1)	22 (34.9)	0.980
Outcomes				
Postoperative atrial fibrillation	53 (11.0)	48 (11.5)	5 (7.9)	0.519
In-hospital mortality	17 (3.5)	14 (3.3)	3 (4.8)	0.476

*OE* open esophagectomy, *MIE* minimally invasive esophagectomy, *BMI* body mass index, *DM* diabetes mellitus, *HTN* hypertension, *HR* heart rate, *LVEF* left ventricular ejection fraction

After adjusting for confounders, MIE was significantly associated with lower POAF risk compared to OE (adjusted odds ratio [aOR] = 0.185, 95% confidence interval [CI] 0.039–0.887, *P* = 0.035). Older patients (aOR for age = 6.194, 95% CI 2.035–18.849, *P* = 0.001) and patients receiving McKeown (aOR = 2.742, 95% CI 1.075–6.990, *P* = 0.035) had a higher risk of POAF compared to their counterparts (Table 2). In sensitivity analysis with adjustment of complications, similar results were found: compared to OE, MIE was negatively associated with POAF (aOR = 0.186, 95% CI 0.040–0.876, *P* = 0.033). The aOR for the complications were 1.361 (0.305–6.078, *P* = 0.686) for anastomotic leak, 2.324 (0.385–14.044, *P* = 0.358) for sepsis, 1.117 (0.181–6.894, *P* = 0.905) for pneumonia, and 0.789 (0.372–1.670, *P* = 0.535) for acute kidney injury (Table S1).

**Table 2:** Adjusted odds ratio of postoperative atrial fibrillation

Variables	Postoperative atrial fibrillation		
	OR	*P* value	95% CI
Procedure			
MIE vs. OE	0.185	0.035	0.039, 0.887
Demographics			
Age ≥ 60 vs. < 60	6.194	0.001	2.035, 18.849
Female vs. male	0.395	0.397	0.046, 3.398
BMI: ≥ 25 vs. < 25	1.301	0.542	0.559, 3.028
HR			
< 51–74 vs. ≤ 51	0.637	0.383	0.232, 1.754
< 74–99 vs. ≤ 51	0.518	0.238	0.174, 1.543
> 99 vs. ≤ 51	1.939	0.178	0.739, 5.087
LVEF (%)			
< 59–62 vs. ≤ 59	0.442	0.105	0.165, 1.185
< 62–65 vs. ≤ 59	0.465	0.135	0.170, 1.268
> 65 vs. ≤ 59	0.719	0.506	0.272, 1.900
Comorbidity			
DM: yes vs. no	1.977	0.149	0.783, 4.993
HTN: yes vs. no	0.603	0.244	0.258, 1.413
Medication			
β-Blocker use: yes vs. no	2.157	0.250	0.582, 8.000
Diuretics use: yes vs. no	0.364	0.232	0.069, 1.911
Neoadjuvant therapy: yes vs. no	0.705	0.523	0.242, 2.059
Pathologic stage of cancer			
Stages I and II vs. stage 0	0.427	0.126	0.144, 1.269
Incision			
McKeown vs. Ivor Lewis	2.742	0.035	1.075, 6.990
Transhiatal vs. Ivor Lewis	1.975	0.595	0.161, 24.259

*OR* odds ratio, *CI* confidence interval, *OE* open esophagectomy, *MIE* minimally invasive esophagectomy, *BMI* body mass index, *DM* diabetes mellitus, *HTN* hypertension, *HR* heart rate, *LVEF* left ventricular ejection fraction

No statistically significant associations between MIE and in-hospital mortality were found (aOR = 0.709, 95% CI 0.114–4.409, *P* = 0.712) (Table 3). Having peri-operative complications of pneumonia (aOR = 48.763, 95% CI 3.716–639.874, *P* = 0.003) was associated with increased odds of in-hospital mortality (Table 3).

**Table 3:** Adjusted odds ratio of in-hospital mortality

Variables	In-hospital mortality		
	OR	*P* value	95% CI
Procedure			
MIE vs. OE	0.709	0.712	0.114, 4.409
Demographics			
Age: ≥ 60 vs. < 60	0.773	0.713	0.196, 3.048
Female vs. male	5.140	0.145	0.570, 46.341
BMI: ≥ 25 vs. < 25	0.246	0.159	0.035, 1.731
Comorbidity			
DM: yes vs. no	1.596	0.573	0.314, 8.113
HTN: yes vs. no	0.430	0.314	0.083, 2.225
Medication			
β-Blocker use: yes vs. no	8.457	0.118	0.582, 122.884
Diuretics use: yes vs. no	1.142	0.942	0.032, 40.809
Neoadjuvant therapy: yes vs. no	0.381	0.303	0.061, 2.391
Pathologic stage of cancer			
Stages I and II vs. stage 0	0.239	0.147	0.034, 1.655
Incision			
McKeown vs. Ivor Lewis	2.091	0.300	0.519, 8.425
Transhiatal vs. Ivor Lewis	76.196	0.013	2.537, 2288.307
Complication			
Pneumonia: yes vs. no	48.763	0.003	3.716, 639.874
Anastomotic leak: yes vs. no	2.299	0.288	0.496, 10.661
Sepsis: yes vs. no	1.451	0.742	0.158, 13.322
Acute kidney injury: yes vs. no	1.663	0.427	0.474, 5.841

*OR* odds ratio, *CI* confidence interval, *OE* open esophagectomy, *MIE* minimally invasive esophagectomy, *BMI* body mass index, *DM* diabetes mellitus, *HTN* hypertension

## Discussion

With the increasing use of MIE, it is urgent to understand the POAF risk after MIE among patients with esophageal cancer. Using hospital-based data, we found that MIE is associated with decreased risk of POAF. However, the association between MIE and in-hospital mortality was not significant.

A few previous studies investigated POAF risk after MIE, but results remain inconsistent [17, 36, 37]. A laparoscopic technique associated with a reduced POAF was found in an early study with patients undergoing foregut surgery [36] (OR = 0.09, 95% CI 0.01–0.95, *P* = 0.04). However, research in Creighton University showed that minimally invasive transthoracic esophagectomy was not associated with a lower risk of POAF (40.5% in OE vs. 59.5% in MIE, *P* = 0.34) [17]. A study conducted in University Medical Center (UMC) Utrecht revealed a marginally significant association (*P* = 0.075) [37]. In this study, after adjusted potential confounders, we found a negative association between MIE and POAF. The possible explanation of reduction in POAF noted with MIE may be due to the minimization of trauma leading to a status of decrease in inflammation [38, 39].

Previous researches identified an interactive effect of POAF and perioperative inflammatory complications such as sepsis and pneumonia [40, 41]. Since the sequence of POAF and these complications cannot be determined in the dataset, we did not adjust for these complications in the multivariable analysis to avoid over-adjustment. However, in a sensitivity analysis adjusting for anastomotic leak, sepsis, pneumonia, and acute kidney injury, the coefficients of these covariates were not significant.

Two additional predictors for POAF in this study were age and type of incision. The positive association between age and POAF was consistent with previous studies [40, 42, 43]. The increased POAF risk for older patients could be due to the acceleration of age-dependent fibrotic changes in Pgc-1β−/− hearts. The latest study made a comparison between Ivor Lewis and McKeown esophagectomy [44], showing that McKeown esophagectomy was associated with more incisional surgical site infections and anastomotic leak, which may justify the positive association between McKeown procedure and POAF.

In short-term survival, inconsistency was found in previous studies [11, 20–22]. MIE was found associated with lower in-hospital mortality in a Japanese Inpatient Database (1.2% vs. 1.7%, *P* = 0.048) [20] and from a meta-analysis (OR = 0.668, 95% CI 0.539–0.827, *P* < 0.05) [11]. However, a multicenter, open-label, randomized controlled trial showed an insignificant relationship between procedures and mortality [21]. In our study, the in-hospital mortality of MIE and OE was not significantly different, which suggests that MIE may reduce the risk of POAF, but the advantage did not extend to short-term survival.

Despite the strengths of hospital-based large sample size and short-term follow-up, this study has two limitations. The first one is generalizability. This study was conducted in an advanced comprehensive medical care center in a developed country. It needs to be cautious to apply the results from this study to other community healthcare settings. Another limitation is the lack of preoperative assessment of left atrial. The study showed that reduced left atrial emptying fraction was associated with the development of POAF [45]. Thus, left atrial mechanical dysfunction may contribute to the risk stratifying of POAF before esophagectomy.

In future studies, assessment of inflammatory characteristics will be helpful to understand the potential mechanism of a lower risk of POAF in MIE. Given the decreased POAF risk from MIE, the future use of new robot-assisted esophagectomy is promising. Additionally, amiodarone and rapid atrial pacing were effective in the prevention of POAF in cardiac surgery [46]. Efforts should be made to carry out further studies examining the development of prophylactic interventions for minimizing the risk of POAF after esophagectomy.

## Conclusion

Compared to OE, MIE is a superior option in reducing POAF risk without a substantial impact on the short-term survival of patients with esophageal cancer.

## Supplementary information

**Additional file 1: Table S1.** Adjusted odds ratio of postoperative atrial fibrillation in sensitivity analysis.

## Data Availability

The de-identified dataset used in this study was obtained from an online platform “FigShare” which is a repository where users can make all of their research outputs available in a citable, shareable, and discoverable manner. Data from FigShare is available under the Creative Commons Attribution License (CCAL) which allows anyone to download, reuse, reprint, modify, distribute, and/or copy data from FigShare [25].
