# Supplementary material for: The role of surgery type in postoperative atrial fibrillation and in-hospital mortality in esophageal cancer patients with preserved left ventricular ejection fraction
Source: World J Surg Oncol. 2020 Sep 11;18:244. doi: 10.1186/s12957-020-02011-6 (PMC7488674; doi:10.1186/s12957-020-02011-6)
Supplement: Supplementary file 1 — Additional file 1: Table S1. Adjusted odds ratio of postoperative atrial fibrillation in sensitivity analysis. [file 12957_2020_2011_MOESM1_ESM.docx]

Table S1 Adjusted odds ratio of postoperative atrial fibrillation in sensitivity analysis.

| Variables | Postoperative Atrial Fibrillation | | |
| --- | --- | --- | --- |
|  | OR | P value | 95% CI |
| Procedure |  |  |  |
| MIE vs. OE | 0.186 | 0.033 | 0.040, 0.876 |
| Demographics |  |  |  |
| Age: ≥60 vs. <60 | 6.464 | 0.001 | 2.052, 20.365 |
| Female vs. Male | 0.378 | 0.400 | 0.039, 3.642 |
| BMI: ≥25 vs. <25 | 1.241 | 0.632 | 0.514, 2.996 |
| HR |  |  |  |
| <51-74 vs. ≤51 | 0.644 | 0.398 | 0.232, 1.789 |
| <74-99 vs. ≤51 | 0.495 | 0.220 | 0.161, 1.522 |
| >99 vs. ≤51 | 1.748 | 0.268 | 0.650, 4.701 |
| LVEF (%) |  |  |  |
| <59-62 vs. ≤59 | 0.411 | 0.092 | 0.146, 1.157 |
| <62-65 vs. ≤59 | 0.457 | 0.134 | 0.164, 1.273 |
| >65 vs. ≤59 | 0.634 | 0.369 | 0.234, 1.716 |
| Comorbidity |  |  |  |
| DM: yes vs. no | 1.872 | 0.198 | 0.720, 4.869 |
| HTN: yes vs. no | 0.690 | 0.402 | 0.289, 1.644 |
| Medication |  |  |  |
| β-blocker use: yes vs. no | 2.054 | 0.292 | 0.539, 7.835 |
| Diuretics use: yes vs. no | 0.372 | 0.261 | 0.066, 2.091 |
| Neoadjuvant therapy: yes vs. no | 0.676 | 0.485 | 0.225, 2.027 |
| Pathologic Stage of Cancer |  |  |  |
| Stage I and II vs. Stage 0 | 0.378 | 0.088 | 0.124, 1.154 |
| Incision |  |  |  |
| McKeown vs. Ivor Lewis | 2.782 | 0.034 | 1.078, 7.182 |
| Transhiatal vs. Ivor Lewis | 1.696 | 0.695 | 0.120, 23.895 |
| Complications |  |  |  |
| Anastomotic leak: yes vs. no | 1.361 | 0.686 | 0.305, 6.078 |
| Pneumonia: yes vs. no | 1.117 | 0.905 | 0.181, 6.894 |
| Sepsis: yes vs. no | 2.324 | 0.358 | 0.385, 14.044 |
| Acute kidney injury: yes vs. no | 0.789 | 0.535 | 0.372, 1.670 |

Abbreviations: OR=Odds Ratio; CI=Confidence Interval; OE=Open Esophagectomy; MIE=Minimally Invasive Esophagectomy; BMI=Body Mass Index; DM= Diabetes Mellitus; HTN=Hypertension; HR=Heart Rate; LVEF=Left Ventricular Ejection Fraction
